# Cuproptosis-Related Risk Score Predicts Prognosis and Characterizes the Tumor Microenvironment in Hepatocellular Carcinoma

**DOI:** 10.3389/fimmu.2022.925618

**Published:** 2022-07-11

**Authors:** Zhen Zhang, Xiangyang Zeng, Yinghua Wu, Yang Liu, Xi Zhang, Zewen Song

**Affiliations:** ^1^ Department of Oncology, The Third Xiangya Hospital of Central South University, Changsha, China; ^2^ Department of Gynecology, The Third Xiangya Hospital of Central South University, Changsha, China; ^3^ Xiangya School of Medicine, Central South University, Changsha, China; ^4^ Department of Pathology, The Third Xiangya Hospital of Central South University, Changsha, China

**Keywords:** Cuproptosis, *FDX1*, hepatocellular carcinoma, prognostic model, tumor microenvironment

## Abstract

**Aims:**

Cuproptosis is a recently identified form of programmed cell death; however, its role in hepatocellular carcinoma (HCC) remains unclear.

**Methods:**

A set of bioinformatic tools was integrated to analyze the expression and prognostic significance of ferredoxin 1 (*FDX1*), the key regulator of cuproptosis. A cuproptosis-related risk score (CRRS) was developed *via* correlation analyses, least absolute shrinkage and selection operator (LASSO) Cox regression, and multivariate Cox regression. The metabolic features, mutation signatures, and immune profile of CRRS-classified HCC patients were investigated, and the role of CRRS in therapy guidance was analyzed.

**Results:**

*FDX1* was significantly downregulated in HCC, and its high expression was associated with longer survival time. HCC patients in the high-CRRS group showed a significantly lower overall survival (OS) and enriched in cancer-related pathways. Mutation analyses revealed that the high-CRRS HCC patients had a high mutational frequency of some tumor suppressors such as tumor protein P53 (*TP53*) and Breast-cancer susceptibility gene 1 (BRCA1)-associated protein 1 (*BAP1*) and a low frequency of catenin beta 1 (*CTNNB1*). Besides, HCC patients with high CRRS showed an increase of protumor immune infiltrates and a high expression of immune checkpoints. Moreover, the area under the curve (AUC) values of CRRS in predicting the efficiency of sorafenib and the non-responsiveness to transcatheter arterial chemoembolization (TACE) in HCC patients reached 0.877 and 0.764, respectively.

**Significance:**

The cuproptosis-related signature is helpful in prognostic prediction and in guiding treatment for HCC patients.

## Introduction

Studies have found that various types of cell death modalities were closely associated with cancer eradication. Badgley et al. (1, 2) concluded that genetically strengthened tumor-selective ferroptosis sensitivity can inhibit the formation and progression of pancreatic cancer in genetically engineered mice. Yu and Liu (3) pointed out the complicated role of necroptosis for both antitumor functions and tumorigenesis and metastasis in cancer. In addition, pyroptosis was found to inhibit the proliferation and metastasis of multiple cancer cells (4, 5). There are also studies that suggested the dual roles that autophagy played in tumor survival (6–8) and metastasis (9, 10). Recently, a novel way of copper-induced cell death distinct from apoptosis, necroptosis, pyroptosis, and ferroptosis has been discovered and termed “cuproptosis” by a study published on *Science* (11). The copper-induced cell death is closely associated with protein lipoylation that is concentrated in the tricarboxylic acid (TCA) cycle for enzymatic function. TCA cycle-active cells have an aggregated lipoylated protein aggregation, loss of Fe-S cluster containing proteins, and induction of heat shock protein 70 (*HSP70*), indicating acute proteotoxic stress (11). They also find that ferredoxin 1 (*FDX1*), an upstream regulator of protein lipoylation, and lipoic acid genes are critical mediators of cuproptosis, suggesting the role of distinguishing genetic divergence in different individuals. While copper plays a fundamental role in numerous biological processes (12, 13), it is controversially suggested that the dyshomeostasis of copper acts prominently in cancer, spanning growth, angiogenesis, and metastasis (14), indicating its potential in developing novel cancer therapies.

The metabolism of copper in liver condition and the development of hepatocarcinoma are still under research. Zhang et al. (15) showed that copper contents are closely related to liver cirrhosis and hepatocellular carcinoma (HCC), and serum copper and ceruloplasmin levels may be used as markers for detection of HCC. The study by Koizumi et al. (16) demonstrated that increased levels of redox-active free copper are likely to be associated with acute hepatitis and, ultimately, hepatocarcinoma. Additionally, the research by Siddiqui et al. (17) suggested that copper oxide nanoparticles induce cytotoxicity and apoptosis in HepG2 cells in dose-dependent manner, which was highly probable to be mediated by reactive oxygen species (ROS) and mitochondrial pathway, respectively. The abovementioned lines of evidence show the role of copper in hepatocarcinogenesis, which indicate that cuproptosis might be closely associated with liver malignancy as well, providing insight into discovering novel therapies for liver cancer.

Although there are already studies that suggested both copper ionophores (18–21) and copper chelators (22–25) as anticancer agents, lack of selectivity is one of the major barriers in the field. Therefore, efforts have been made to resolve this through the conjugation of targeting units with copper ionophores. Also, other possibilities have been raised, such as the application of proionophores or nano-drug delivery system. For instance, Bao et al. (26, 27) have proposed that proionophores activated by cancer cells exploit their susceptibility to ROS. Gaál et al. (28) used a thermosensitive liposomal formulation loaded with copper and neocuproine to observe *in vitro* and *in vivo* toxicity in mice with C26 cancer cells. The future direction for copper ionophores should focus more on targeting certain specific receptors found exclusively in a specific type of tumor cells (14).

Here in our study, we explored the association of the expression of *FDX1* and cancer stages as well as prognosis in HCC. A novel index, based on *FDX1* and its related genes, was developed and named “cuproptosis-related risk score” (CRRS) for cuproptosis risk and survival condition. We also analyzed the metabolic characteristics, genetic mutation landscape, and immune-related components of cuproptosis in different risk groups of HCC patients, and the results indicated that the two groups had obvious distinction in all these aspects. The evaluation of our predictive typing model showed great potential in providing guidance for hepatocarcinoma typing and therapy of CRRS-sensitive patients, demonstrating its clinical value and significance.

## Materials and Methods

### Public Data Acquisition and Processing

The gene expression data, phenotype data, and corresponding survival information (if available) of liver hepatocellular carcinoma (LIHC) of The Cancer Genome Atlas (TCGA), the liver cancer project (code: LIRI_JP) of the International Cancer Genome Consortium (ICGC), GSE64041, GSE14520/GPL3921, GSE76427, GSE104580, GSE109211, and GSE25097 were downloaded from public databases and were processed as reported in our previous publications (29–31). The single-cell analysis of *FDX1* in hepatocellular carcinoma was conducted in the Tumor Immune Single-Cell Hub (TISCH) database (http://tisch.comp-genomics.org/). The mutation data of TCGA-LIHC cohort (workflow type: varScan2 variant aggregation and masking) were downloaded, processed, and visualized as reported in our previous study (29). Since these data were all available online with usage allowance, an extra ethical approval was not necessary.

### Development of Cuproptosis-Related Risk Score (CRRS)

*FDX1* and its related genes were input into the least absolute shrinkage and selection operator (LASSO) Cox regression analysis in the GSE14520 dataset, and the analysis was conducted via the glmnet package in R. The generated 4 crucial genes were further undergoing multivariate Cox regression analysis and a score of each sample was obtained by multiplying the gene expression value of each crucial gene and its corresponding coefficient. Namely, score = =- 0.13681 * *CAT* - 0.07452 * *EHHADH* - 0.09026 * *ALDH5A1* - 0.12120 * *SLC27A5*. To facilitation comparison across different cohorts, CRRS was calculated with the formula reported in our previous study, namely, CRRS = (score-Min)/absolute(Max) (32).

### Enrichment Analysis

Gene set enrichment analysis (GSEA) of CRRS-based classification of HCC patients and Gene Ontology (GO) and Kyoto Encyclopedia of Genes and Genomes (KEGG) enrichment analysis of *FDX1*-related genes were conducted as reported in our previous studies (29, 31).

### Immune Profile Analysis

The immune score and stromal score of each sample in TCGA-LIHC cohort was calculated by the “estimate” package in R (33). The proportion of the 22 types of immune cells in the tumor microenvironment (TME) of each sample was evaluated *via* the CIBERSORT algorithm in R software (34).

### Statistical Analysis

The data analyses and visualization were conducted in R (version 4.1.1), and the following packages were used: “tidyverse,” “maftools,” “GEOquery,” “limma,” “survival,” “survminer,” “dplyr,” “plyr,” “survivalROC,” “timeROC,” “ggplot2,” “ggplotify,” “cowplot,” “Hmisc,” “gridExtra,” “GSVA,” “clusterProfiler,” “corrplot,” “VennDiagram,” and “pheatmap.” The Wilcoxon test was used for comparison of data between two groups, whereas the ANOVA test was for comparison of data among three groups. For prognosis analysis, the HCC patients in the training dataset were divided into two subgroups based on the optimal cutoff value of a marker determined by the “survminer” package in R (29, 35). The ratio of high-risk patients to low-risk ones in the training dataset was then applied to the validating datasets. The Kaplan–Meier method was used for prognosis analysis. Pearson method was used for correlation analysis. The tumor mutation burden (TMB) and the mutant-allele tumor heterogeneity (MATH) score were calculated by the package “maftools” in R. A p value <0.05 was considered statistically significant (* *p* < 0.05; ** *p* < 0.01; *** *p* < 0.001; **** *p* < 0.0001).

## Results

### Ferredoxin 1 Was Downregulated in Hepatocellular Carcinoma

**Figure 1:**
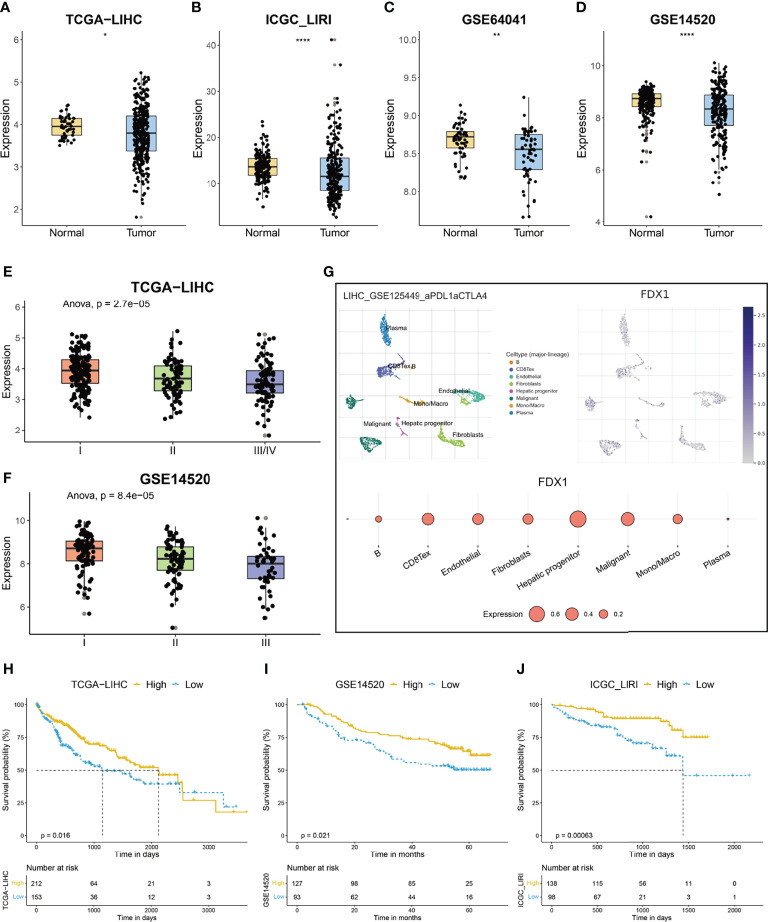
The expression and prognostic significance of ferredoxin 1 (*FDX1*) in hepatocellular carcinoma (HCC). **(A–D)** The box and dot plot showing the expression of *FDX1* between tumor and corresponding normal tissues in TCGA-LIHC **(A)**, ICGC-LIRI **(B)**, GSE64041 **(C)**, and GSE14520 **(D)** cohorts. Wilcoxon test was used for data analyses. **(E, F)** The box and dot plot showing the expression of *FDX1* in HCC patients with different clinical stages in TCGA-LIHC **(E)** and GSE14520 **(F)** cohorts. ANOVA test was used for data analyses. **(G)** The expression of *FDX1* in each type of cell in the GSE125449 dataset. The upper left t-SNE subgraph reveals the distribution of eight types of cells from HCC patients (indicated by colors). The upper right subgraph showed the relative expression of *FDX1* in each cell. The lower subgraph showed the relative expression of *FDX1* in eight types of cells. **(H–J)** The prognostic significance of *FDX1* in TCGA-LIHC **(H)**, GSE14520 **(I)**, and ICGC-LIRI **(J)** cohorts. Based on the optimal cutoff value of *FDX1*, about 58% (212/365) of the HCC patients in TCGA-LIHC were divided into high-*FDX1* subgroup and the remaining patients were in the low-*FDX1* subgroup. In the validating datasets (GSE14520 and ICGC_LIRI), 58% of HCC patients were also classified into the high-*FDX1* subgroup according to the expression of the gene. The Kaplan–Meier method was used for prognosis analysis. The p values were shown as *p < 0.05, **p < 0.01 and ****p < 0.0001.

*FDX1* functions as a key regulator of cuproptosis, as deletion of the gene caused resistance to copper-induced cell death (11). To evaluate the status of cuproptosis in HCC, we first explored the expression of *FDX1* in the disease. *FDX1* had a relatively high expression in the liver among various normal tissues; however, its expression in liver cancers cannot be detected, suggesting a possible downregulation of the gene during the development of HCC (**Supplementary Figures S1A, B**). As shown in **Figures 1A–D** **and** **Supplementary Figures S1C, D**, HCC tissue indeed exhibited a significantly lower expression of *FDX1* when compared with its expression in normal tissue. More importantly, the *FDX1* expression showed a gradient descent as the clinical stage advanced (**Figures 1E, F** **)**. Single-cell RNA sequencing of HCC samples indicated that *FDX1* can be detected in both tumor cells and non-tumor cells like fibroblasts and monocytes (**Figure 1G**), but its expression was predominantly detected in hepatic progenitor cells and malignant cells (**Figure 1G**). When HCC patients were categorized into two groups based on the best cutoff value of the expression of *FDX1* in TCGA-LIHC dataset, about 58% (212/365) of them were labeled as high-*FDX1* and exhibited a significantly longer survival time than that of the remaining low-*FDX1* HCC patients (**Figure 1H**, p = 0.016). Similarly, when 58% of HCC patients were classified into the high-*FDX1* group in the GSE14520 and ICGC-LIRI datasets, according to the expression of *FDX1*, they also had a significantly better prognosis (**Figures 1I, J**; p = 0.021 and p = 0.00063, respectively).

### Construction and Validation of Cuproptosis-Related Risk Score

**Figure 2:**
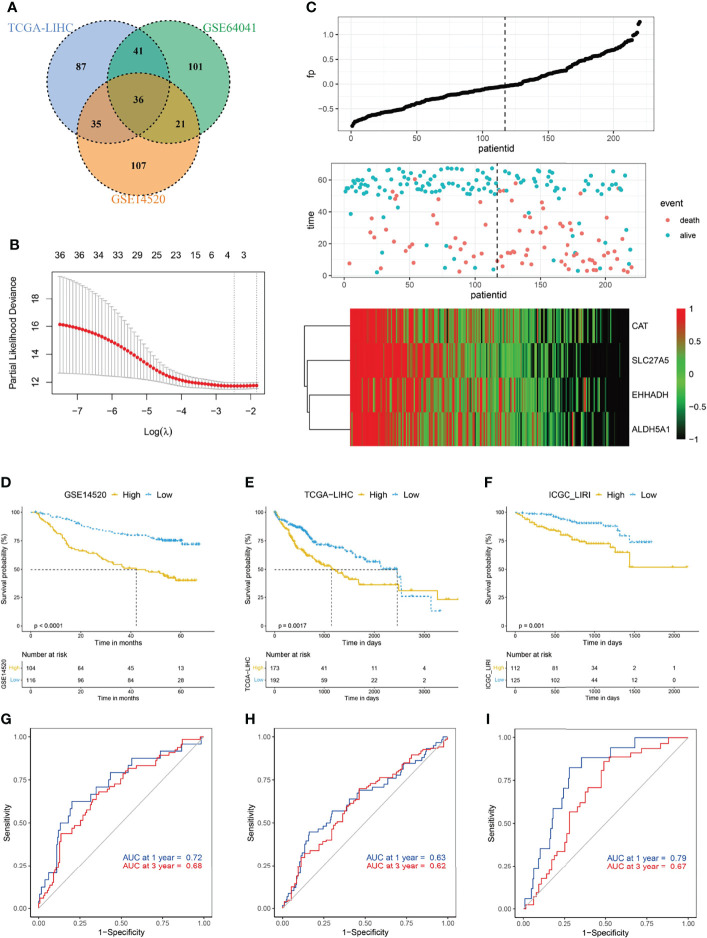
Construction of cuproptosis-related risk score (CRRS). **(A)** Venn diagram indicating the 36 *FDX1*-related genes identified in all three cohorts. **(B)** The LASSO Cox regression model was constructed from *FDX1* and its related genes. The tuning parameter (λ) was calculated based on the partial likelihood deviance with 10-fold cross validation. An optimal log λ value shown by the vertical black dot-lines in the plots. The four signature genes were identified according to the best fit profile. **(C)** The distribution and optimal cutoff value of CRRS, the OS status of each sample, and the expression value of the four crucial genes in the GSE14520 dataset. **(D–F)** The prognostic significance of CRRS in GSE14520 **(D)**, TCGA-LIHC **(E)**, and ICGC-LIRI **(F)** cohorts. About 47.3% (104/220) of the HCC patients in the GSE14520 were divided into high-CRRS subgroup and the remaining patients were in the low-CRRS subgroup based on the optimal cutoff value of the CRRS. In the validating datasets (TCGA-LIHC and ICGC_LIRI), 47.3% of HCC patients were also classified into the high-CRRS subgroup according to the value of CRRS. The Kaplan–Meier method was used for prognosis analysis. **(G–I)** Time-dependent ROC analyses of the CRRS regarding the OS and survival status in the GSE14520 **(G)**, TCGA-LIHC **(H)**, and ICGC-LIRI **(I)** cohorts.

*FDX1* encodes a reductase that reduces Cu^2+^ to a more toxic Cu^1+^; moreover, *FDX1* regulates protein lipoylation and facilitates the formation of oligomerization of lipoylated dihydrolipoamide S-acetyltransferase (DLAT) and subsequent induction of proteotoxic stress (11). Given the pivotal role of *FDX1* in cuproptosis, we hypothesized that an *FDX1*-related signature might help to evaluate the occurrence of this copper-induced cell death in HCC. We first conducted a correlation analysis, and 36 genes were identified among the top 200 genes, based on the coefficient, from three datasets (**Figure 2A**). Protein lipoylation occurs on only four enzymes that regulate carbon entry points to the TCA cycle. Accordingly, GO analysis of these 36 *FDX1*-related genes showed that they were enriched in TCA cycle-related molecular function and biological processes, such as long-chain fatty acid-CoA ligase activity, electron transfer activity, and carboxylic acid catabolic process (**Supplementary Figure S2A**). KEGG analysis showed that these genes were enriched in carbon metabolism, pyruvate metabolism, and so on (**Supplementary Figure S2B**).

*FDX1* and its related genes were then input into a LASSO regression model and generated four crucial genes, namely, catalase (*CAT*), solute carrier family 27A (*SLC27A*), enoyl-CoA hydratase and 3-hydroxyacyl CoA dehydrogenase (*EHHADH*), and aldehyde dehydrogenase 5 family member A1 (*ALDH5A1*) (**Figures 2B, C**; **Supplementary Figure S2C**). CRRS was then constructed by the way demonstrated in the *Materials and Methods* section. Based on the best cutoff value of CRRS in the GSE14520 dataset (**Supplementary Figure S2D**), about 47.3% HCC patients were classified into the high-CRRS group, and these patients had considerably shorter survival time when compared with the remaining patients in the low-CRRS group (**Figure 2D**). The area under the curve (AUC) values of CRRS in predicting the overall survival (OS) time were 0.72 at 1 year and 0.68 at 3 years (**Figure 2G**). In the two external validating cohorts (TCGA-LIHC and ICGC-LIRI datasets), when 47.3% HCC patients were categorized into high-CRRS group based on the value of CRRS in each cohort, they also had significantly shorter OS (**Figures 2E, F**). The AUC values of CRRS in TCGA-LIHC were 0.63 for 1 year and 0.62 for 3 years and were 0.79 for 1 year and 0.67 for 3 years in the ICGC-LIRI dataset (**Figures 2H, I** **)**. Furthermore, HCC patients with a high CRRS in the GSE14520 and TCGA-LIHC datasets also exhibited significantly shorter progression-free survival (PFS; **Supplementary Figures S2E, F**).

We further investigated the relationship between CRRS and clinicopathological features of HCC patients. As shown in **Table 1**, no consistent difference was found between HCC patients with high and low CRRS with regard to age, gender, or fibrosis/cirrhosis state. However, a significantly higher percentage of HCC patients in the high-CRRS group exhibited a high level of alpha fetoprotein (AFP) (>300 ng/ml, p < 0.001), were at an advanced TNM stage (stage III or IV, p < 0.001 and p = 0.003, respectively) and Barcelona Clinic Liver Cancer (BCLC) stage (stage B or C, p < 0.001), and had higher histologic grades (G3 or G4, p < 0.001). Taken together, these results indicated that a high CRRS value was associated with worse clinicopathological features of HCC patients.

**Table 1:** Relationships between cuproptosis-related risk score (CRRS) and clinicopathological features of clinicopathological features of hepatocellular carcinoma (HCC) patients.

		GSE14520			TCGA		
		Low CRRS	High CRRS	p value	Low CRRS	High CRRS	p value
Age	<60	87	91	0.0249	80	85	0.171
	≥60	29	13		112	88	
Gender	Men	102	88	0.556	146	100	<0.001
	Women	14	16		46	73	
Fibrosis/Cirrhosis	No	12	6	0.229	46	28	0.883
	Yes	103	98		82	53	
AFP (ng/ml)	≤300	82	36	<0.001	132	80	<0.001
	>300	32	67		16	48	
Stage	I/II	100	68	<0.001	145	109	0.003
	III/IV	13	35		33	54	
BCLC	0/A	100	68	<0.001			
	B/C	15	35				
Child_pugh	A				125	91	0.823
	B/C				12	10	
Histologic_grade	G1+G2				140	90	<0.001
	G3+G4				48	82	

AFP, alpha fetoprotein; BCLC, Barcelona Clinic Liver Cancer.

**Figure 3:**
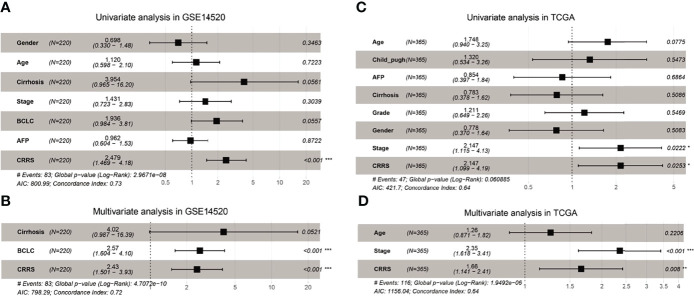
CRRS was an independent prognostic predictor for HCC patients. **(A, B)** Results of the univariate **(A)** and multivariate **(B)** Cox regression analyses regarding OS in the GSE14520 cohort. **(C, D)** Results of the univariate **(C)** and multivariate **(D)** Cox regression analyses regarding OS in TCGA-LIHC cohort.

To determine whether CRRS could serve as an independent prognostic predictor of OS, the clinicopathological features and CRRS were first input into a univariate Cox regression analysis. As shown in **Figures 3A, C**, CRRS was found to be significantly associated with OS in both GSE14520 [Hazard ratio (HR) = 2.479, 95% CI = 1.469–4.19, p < 0.001] and TCGA datasets (HR = 2.147, 95% CI = 1.099–4.19, p = 0.0253). Then, CRRS, cirrhosis, and BCLC stage in the GSE14520 dataset and CRRS, clinical stage, and age in TCGA-LIHC dataset were subjected to a multivariate Cox regression analysis, which revealed that CRRS remained an independent prognostic predictor after correction for other confounding factors (p < 0.001 and p = 0.008, respectively; **Figures 3B, D**).

In addition, HCC patients in the high-CRRS group had a significantly decreased expression of several pro-cuproptosis genes like *FDX1*, dihydrolipoamide dehydrogenase (*DLD*), and pyruvate dehydrogenase E1 subunit alpha 1 (*PDHA1*) while showing a considerably upregulated expression of cyclin-dependent kinase inhibitor 2A (*CDKN2A*), an anti-cuproptosis gene (**Supplementary Figure S2E**).

### Metabolic Features of Cuproptosis-Related Risk Score-Based Classification

**Figure 4:**
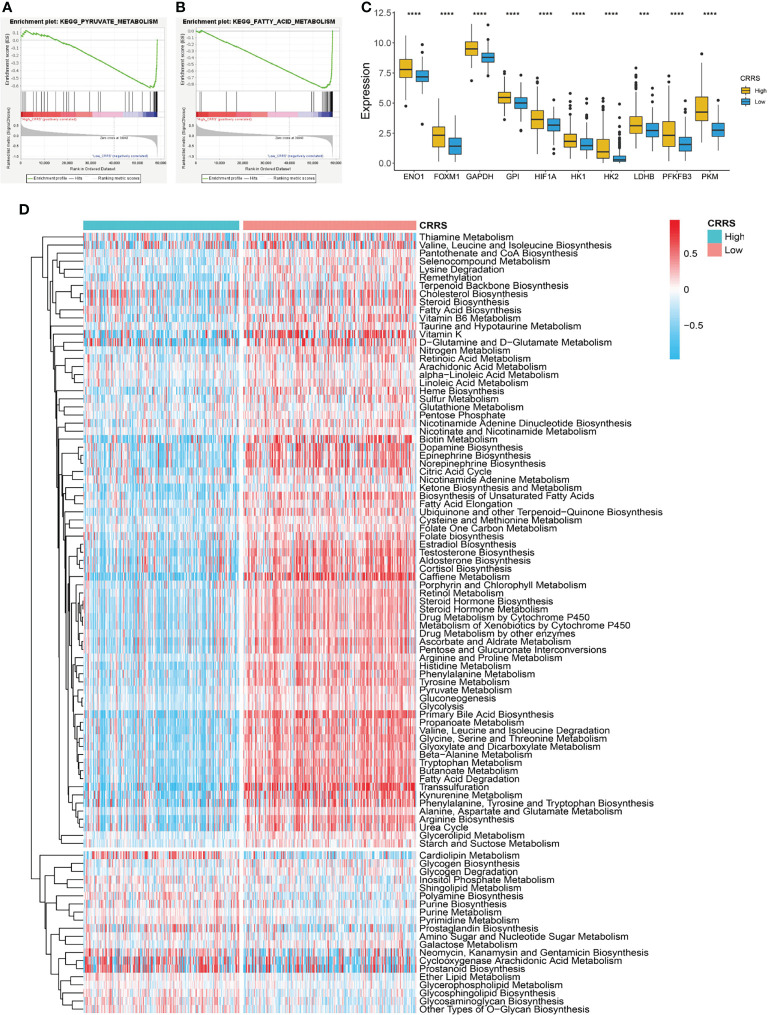
Metabolic features of CRRS-based HCC groups. **(A, B)** GSEA of the low-CRRS group in TCGA-LIHC cohort. **(C)** Boxplot showing the expression of glycolysis-related genes in the HCC patients with low or high CRRS. Wilcoxon test was used for data analyses. **(D)** Heatmap of the enrichment score of metabolism-related pathways in the HCC patients from the low- or high-CRRS subgroups. The p values were shown as ****p < 0.0001.

Cells undergoing mitochondrial respiration are more vulnerable to cuproptosis than those relying on glycolysis. GSEA revealed that HCC patients in the low-CRRS group showed an enrichment in metabolism-related pathways such as fatty acid metabolism, pyruvate metabolism, and citrate cycle (TCA cycle) (**Figures 4A, B**; **Supplementary Table S1**), whereas patients in the high-CRRS group showed cancer-related pathways such as WNT signaling, Notch pathway, and Hedgehog signaling (**Supplementary Figures S3A, B**; **Supplementary Table S2**). Many genes encoding enzymes of the glycolytic pathway, such as hexokinase 2 (*HK2*), pyruvate kinase M1/2 (*PKM*), lactate dehydrogenase B (*LDHB*), and glyceraldehyde-3-phosphate dehydrogenase (*GAPDH*), were all significantly upregulated in HCC patients with high CRRS (**Figure 4C**) (36). Forkhead box M1 (*FOXM1*), a known transcription factor promoting glycolysis in some cancers by binding to the promoters of glycolytic enzyme genes including lactate dehydrogenase A (*LDHA*), hexokinase 1 (*HK1*), glucose transporters (*GLUTs*), and 6-phosphofructo-2-kinase/fructose-2,6-biphosphatase 3 (*PFKFB3*), was also increased in expression in patients in the high-CRRS group (**Figure 4C**) (37). Hypoxia-inducible factor-1 (*HIF-1*) determines the consumption of glucose *via* oxidation or glycolysis, and a sustainable increase of *HIF-1*α would enhance aerobic glycolysis in tumor cells (38). We noticed that *HIF1A* was also elevated in patients with high CRRS (**Figure 4C**). Consistent with the GSEA results, quantification of metabolism processes using a set of genes identified by Yang et al. (39) indicated that the low-CRRS HCC patients were predominantly enriched in citric acid cycle-related pathways, whereas high-CRRS HCC patients were enriched in ether lipid metabolism, purine metabolism, and pyrimidine metabolism (**Figure 4D**).

### Mutation Landscape of Cuproptosis-Related Risk Score-Based Classification

**Figure 5:**
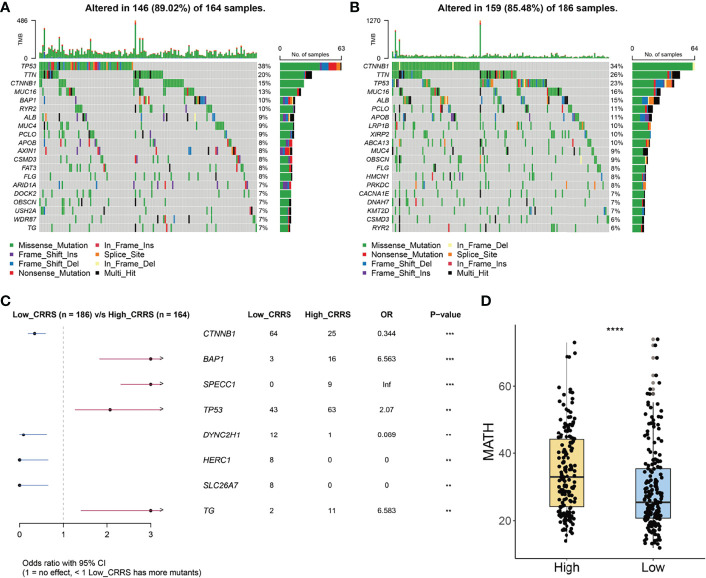
Mutation signatures of CRRS-based HCC groups. **(A, B)** Oncoplots of the mutated genes in the HCC patients from high-CRRS **(A)** and low-CRRS **(B)** subgroups. The mutation types of genes were marked by different colors. **(C)** Forest plot of the differentially mutated genes between HCC patients from high- and low-CRRS subgroups. **(D)** Box and dot plot showing the MATH scores of HCC patients from high- and low-CRRS subgroups. Wilcoxon test was used for data analyses. The p values were shown as ****p < 0.0001.

We further investigated the mutation profile of CRRS-stratified HCC patients. Although no difference was observed in tumor mutation burden between the two groups (**Supplementary Figure S3C**), these patients exhibited different mutation signatures. As shown in **Figures 5A, B**, the top 5 genes with the highest mutant frequency in the low-CRRS group were catenin beta 1 (*CTNNB1*; 34%); titin (*TTN*; 26%); tumor protein P53 (*TP53*; 23%); mucin 16, cell surface associated (*MUC16*; 16%); and albumin (*ALB*; 15%), whereas those were *TP53* (38%), *TTN* (20%), *CTNNB1* (15%), *MUC16* (13%), and BRCA1-associated protein 1 (*BAP1*; 10%) in the high-CRRS group.

Notably, 64 patients in the low-CRRS group and 25 patients in the high-CRRS group exhibited *CTNNB1* mutation [odds ratio (OR) = 0.344, p < 0.001, **Figure 5C**]. Furthermore, mutation of dynein cytoplasmic 2 heavy chain 1 (*DYNC2H1*), HECT and RLD domain-containing E3 ubiquitin protein ligase family member 1 (*HERC1*), and solute carrier family 26 member 7 (*SLC26A7*) was easier to be detected in HCC patients from the low-CRRS group (p < 0.01, **Figure 5C**), and *BAP1*, sperm antigen with calponin homology and coiled-coil domains 1 (*SPECC1*), *TP53*, and thyroglobulin (TG) had a significantly higher mutant frequency in HCC patients from the high-CRRS group (p < 0.01, **Figure 5C**). MATH score, a reflection of tumor heterogeneity, was found to be significantly increased in the high-CRRS patients (**Figure 5D**).

### Immune Landscape of Cuproptosis-Related Risk Score-Based Classification

**Figure 6:**
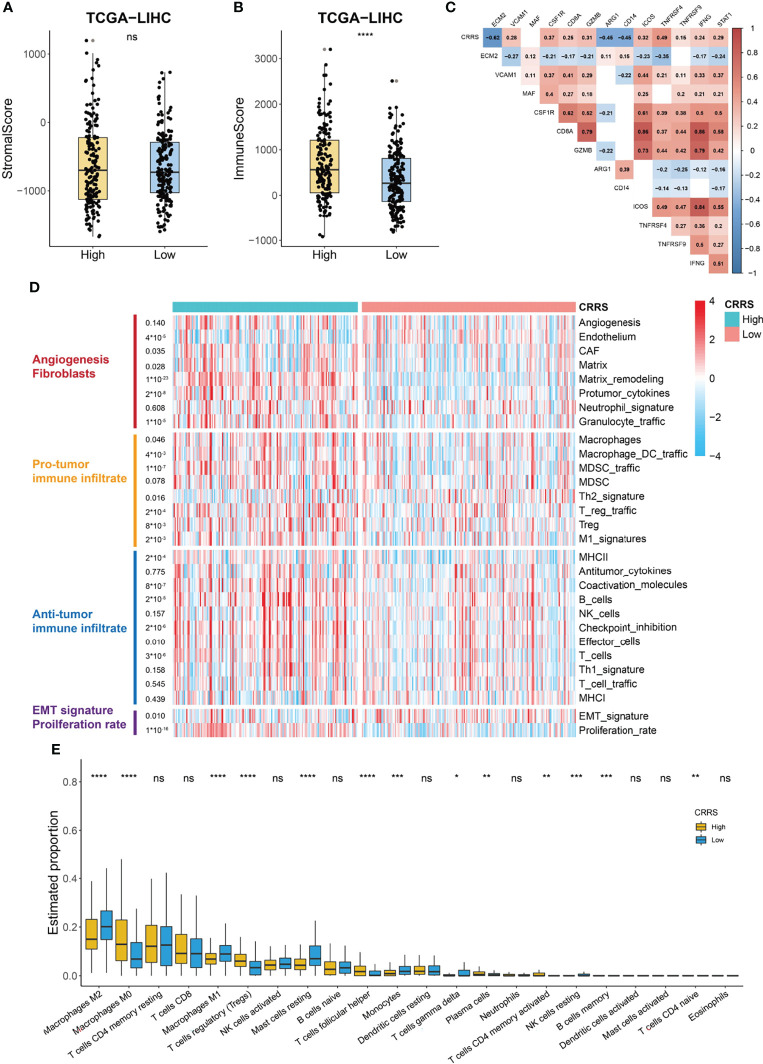
Immune profile of CRRS-based HCC groups. **(A, B)** Box and dot plot showing stromal score **(A)** and immune score **(B)** of HCC patients from high- and low-CRRS subgroups. Wilcoxon test was used for data analyses. **(C)** Association between CRRS and stroma or immune-related markers. Data were analyzed using Pearson correlation analysis. **(D)** Heatmap of the enrichment score of 29 Fregs in HCC patients from the low- or high-CRRS subgroups. **(E)** The boxplot showing the proportion of the 22 types of immune cells in HCC patients from the low- or high-CRRS subgroups. The p values were shown as *p < 0.05, **p < 0.01, ***p < 0.001, and ****p < 0.0001. ns for not significant.

The TME consists of a variety of nontumor cells including immune cells and stromal cells such as endothelial cells and fibroblasts and profoundly impacts cancer growth and invasion (40). We noticed that stromal score showed no difference between the high- and low-CRRS HCC patients (p = 0.93, **Figure 6A**); however, the immune score of high-CRRS patients was significantly higher than that of low-CRRS patients (p < 0.0001, **Figure 6B**), suggesting a higher infiltration of immune cells in the high-CRRS group. Extracellular matrix protein 2 (*ECM2*), one of the stromal signatures, had a strong negative correlation with CRRS (r = -0.62, **Figure 6C**), while some other stromal signatures such as intercellular adhesion molecule 1 (*ICAM1*) and colony-stimulating factor 1 receptor (*CSF1R*) showed a significantly positive correlation with CRRS (**Figure 6C**). CD8a molecule (*CD8A*) and granzyme B (*GZMB*), two phenotypic and functional markers of T cells, showed a positive correlation with CRRS, whereas a significantly negative correlation existed between CRRS and two markers of myeloid cells, namely, arginase 1 (*ARG1*) and *CD14* (**Figure 6C**). CRRS also showed a significantly positive correlation with immune-active biomarkers [inducible T cell costimulator (*ICOS*), TNF receptor superfamily member 4 (*TNFRSF4*), and TNF receptor superfamily member 9 *TNFRSF9*] and IFNγ signatures (*IFNG* and *STAT1*) (**Figure 6C**).

The above analyses indicated that CRRS might have a different correlation with components of the TME. Bagaev et al. (41) proposed a list of 29 functional gene expression signatures (Fges) representing cellular and functional TME properties. Using the data from this publication, we noticed that the high-CRRS HCC patients were characterized by the elevated expression of Fregs associated with cancer-associated fibroblast (CAF) activation, protumor immune infiltration, and a higher proliferation rate (**Figure 6D**). Some antitumor immune infiltrates, such as coactivation molecules, B cells, checkpoint inhibition, and T cells, also showed a significantly higher enrichment in the high-CRRS group, whereas no difference was observed in the enrichment of the remaining antitumor immune infiltrate-related terms like antitumor cytokines (**Figure 6D**). We further evaluated the infiltration level of immune cells in TCGA-LIHC cohort using the CIBERSORT algorithm (**Figure 6E**). Patients in the two groups exhibited no difference in the infiltration of most antitumoral immune cells, including CD8+ T cells, activated NK cells, activated memory CD4+ T cells, neutrophils, and activated dendritic cells, while patients in the high-CRRS group showed a higher fraction of M0 macrophages (p < 0.0001), regulatory T cells (p < 0.001), and follicular helper T cells (p < 0.0001) (**Figure 6E**). Moreover, a higher level of M2 macrophages (p < 0.01), M1 macrophages (p < 0.0001), resting mast cells (p < 0.0001), and monocytes (p < 0.001) was observed in HCC patients from the low-CRRS group (**Figure 6E**).

### Guidance of Cuproptosis-Related Risk Score in Tumor Therapy

**Figure 7:**
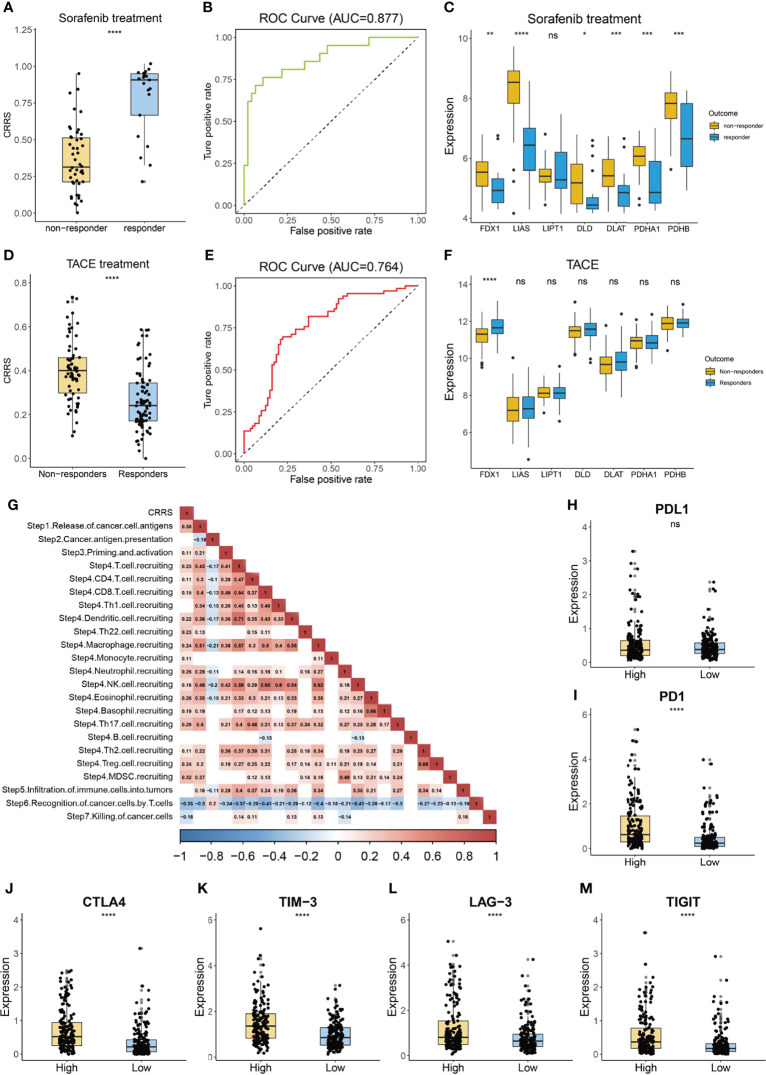
Guidance of CRRS in the therapy for HCC patients. **(A)** The box and dot plot showing the value of CRRS between responders and non-responders to sorafenib. Wilcoxon test was used for data analyses. **(B)** The AUC value of CRRS in predicting the efficiency of sorafenib in HCC patients. **(C)** The boxplot showing the expression of cuproptosis-related genes in the responders and non-responders to sorafenib. Wilcoxon test was used for data analyses. **(D)** The box and dot plot showing the value of CRRS between responders and non-responders to TACE. Wilcoxon test was used for data analyses. **(E)** The AUC value of CRRS in predicting efficiency of TACE in HCC patients. **(F)** The boxplot showing the expression of cuproptosis-related genes in responders and non-responders to TACE. Wilcoxon test was used for data analyses. **(G)** Correlation between CRRS and immune activity scores of each step of the Cancer-Immunity Cycle. Data were analyzed using Pearson correlation analysis. **(H–M)** The box and dot plot showing the expression of *PDL1* **(H)**, *PD-1* **(I)**, *CTLA-4* **(J)**, *TIM-3* **(K)**, *LAG-3* **(L)**, and *TIGIT* **(M)** between HCC patients from the low- or high-CRRS subgroups. Wilcoxon test was used for data analyses. The p values were shown as *p < 0.05, **p < 0.01, ***p < 0.001, and ****p < 0.0001. ns for not significant.

Sorafenib is the first-line treatment for HCC patients at advanced stage (42). As shown in **Figure 7A**, we found that patients who responded to sorafenib had dramatically lower values of CRRS, and the AUC value of CRRS in predicting the responsiveness to sorafenib reached 0.877 (**Figure 7B**), suggesting that HCC patients with a cuproptosis-resistant status might respond to the sorafeinib (a systematic treatment for patients with advanced hepatocellular carcinoma). Consistently, most anti-cuproptosis genes, such as *FDX1*, *LIAS*, and *PDHB*, had a significantly lower expression in responders than that in non-responders (**Figure 7C**). We also evaluated the relationship between CRRS and responsiveness to transcatheter arterial chemoembolization (TACE), a widely used treatment for HCC patients at stage BCLC A or B (43). Interestingly, patients who did not respond to TACE had significantly higher values of CRRS (p = 3.8e-08, **Figure 7D**), and the AUC value of CRRS in predicting the non-responsiveness of HCC patients to TACE was 0.764 (**Figure 7E**). *FDX1*, the key regulator in the copper ionophore–induced cell death, had a significantly higher expression in patients who responded to TACE (p < 0.0001, **Figure 7F**). We further explored the association of CRRS with immune therapy. Xu et al. (44) divided the anticancer immune response into a series of stepwise events. As shown in **Figure 7G**, CRRS exhibited a significantly positive correlation with the release of cancer cell antigens (step 1), priming and activation (step 3), and recruiting of various types of immune cells (step 4) but showed a significantly negative correlation with the recognition of cancer cells by T cells (step 6) and killing of cancer cells (step 7). In addition, to predict the response of immune checkpoint inhibitors (ICIs), we analyzed the relationship between the expression of immune checkpoints in CRRS-stratified subgroups. As shown in **Figures 7H–M**, although no difference was observed in the expression of programmed cell death 1 ligand 1 (*PDL1*) between the high- and low-CRRS groups, the expression of programmed cell death protein 1 (*PD-1*), cytotoxic T-lymphocyte-associated protein 4 (*CTLA-4*), T cell immunoglobulin and mucin-containing molecule 3 (*TIM-3*), lymphocyte-activation gene 3 (*LAG-3*), and T cell immunoreceptor with Ig and ITIM domains (*TIGIT*) was all significantly higher in the high-CRRS group than that in the low-CRRS group, suggesting that HCC patients with a high CRRS were more likely to benefit from the ICIs.

## Discussion

The liver plays a pivotal role in copper metabolism. Due to the oxidative potential of copper in inducing free radical production, excess storage of copper could lead to cellular damage and even pathological diseases such as Wilson’s disease (45). Liver cirrhosis, a well-known cause for HCC, showed an accumulation of copper when compared with healthy liver (46). Thus, we hypothesized that HCC cells might develop a strategy in attenuating the toxicity of the increased concentration of copper and avoiding the incidence of recent proposed cuproptosis. Knockout of *FDX1*, a key regulator of copper ionophore–induced cell death, results in complete loss of protein lipoylation and resistance to cuproptosis (11). HCC samples had significantly lower expression of *FDX1* than corresponding normal liver tissues (**Figures 1A–D**; **Supplementary Figures S1D, E**), suggesting a certain resistance to cuproptosis. In addition, HCC patients with a lower expression of *FDX1* is associated with shorter survival time (**Figures 1H–J**) probably due to the gain of survival advantage of these tumor cells by resisting against copper-induced toxicity. Except *FDX1*, the human genome has another ferredoxin, *FDX2*. Although both *FDX1* and *FDX2* participate in the transfer of electrons from nicotinamide adenine dinucleotide phosphate (NADPH) to cytochrome P450 for biogenesis of Fe-S clusters, they have different roles in HCC (47). *FDX2*, instead of *FDX1*, is involved in ferredoxin reductase (*FDXR*)-regulated iron homeostasis and might contribute to p53-dependent ferroptosis (48).

Given the pivotal role of *FDX1* in cuproptosis, we developed a CRRS by selecting crucial genes of *FDX1*-related genes *via* LASSO regression and multivariate Cox analysis. All of the four genes had decreased expression in HCC patients with high CRRS (**Figure 2C**). *CAT* encodes a heme enzyme, catalase, which presents in the peroxisome of nearly all aerobic cells and functions against the destructive effects of ROS, facilitating oxidative cellular metabolism (49). *SLC27A5* participates in fatty acid transport and bile acid metabolism and functions as a tumor suppressor in HCC. Its downregulation in HCC has been demonstrated to contribute to tumor invasion and progression by promoting epithelial–mesenchymal transition (EMT) and by increasing polyunsaturated lipids and subsequent ROS production as well as lipid peroxidation *via* activating NRF2/TXNRD1 pathway (50, 51). *EHHADH* plays a prominent role in the peroxisomal beta-oxidation of long-chain fatty acids (52). A recent study found that downregulation of *EHHADH* resulted in accumulation of the toxic metabolite dodecanedioic acid (DDDA) and subsequent hepatic necrosis and might play a role in the development of HCC (53). *ALDH5A1* is involved in the metabolism of the neurotransmitter 4-gamma-aminobutyric acid (GABA). Although the role of *ALDH5A1* in HCC is unclear at present, its downregulation in high-grade serous ovarian cancer (HGSOC) is associated with the presence of an EMT gene signature and poor prognosis, implying a role in the migration and invasion of HGSOC (54). Taken together, all of the four crucial genes regulate metabolism-related pathways and are involved in the development and progression of tumor.

HCC patients with high CRRS might exhibit resistance to cuproptosis. Firstly, these patients had decreased expression of pro-cuproptosis genes such as *FDX1*, *DLD*, and *PDHA1*, whereas they showed a considerably upregulated expression of *CDKN2A*, an anti-cuproptosis gene (**Supplementary Figure S2E**) (11). Secondly, cells undergoing glycolysis or growing under hypoxia conditions had considerable insensitivity to cuproptosis (11), and patients with high CRRS had significantly higher expression of hypoxia- or glycolysis-related genes than those with low CRRS (**Figure 4C**). Correspondingly, the low-CRRS patients were enriched in citric acid cycle-related pathways (**Figures 4A, B, D** **)**. Probably due to their acquired capability in attenuating copper-induced toxicity, HCC patients with a high CRRS might have an even aggressive phenotype and showed considerably shorter survival time (**Figures 2D–F**).

The combination of ICIs and bevacizumab has shown superiority than sorafenib in unresectable HCC (55, 56). Interestingly, we found that HCC patients with high CRRS exhibited an increased infiltration of protumor immune components, whereas no difference was observed in the fraction of antitumor immune cells such as CD8 T cells and B cells (**Figures 6D, E** **)**. In addition, the expression of major immune checkpoint targets, such as *PD1*, *TIGIT*, and *TIM-3*, was significantly elevated in the high-CRRS group (**Figures 7I–M**), and CRRS had a negative association with the recognition of cancer cells by T cells (**Figure 7G**), further suggesting an immune-suppressive characterization of the high-CRRS group. In addition, recent studies revealed that ferroptosis exerted a complicated impact on the function of tumor-infiltrating immune cells (57–59). CD8 T cells could induce ferroptosis in cancer cells, and the regulated cell death is part of the antitumor mechanism of immunotherapy (57, 59). However, the occurrence of ferroptosis in immune cells, facilitated by receptors like CD36, resulted in impaired antitumor ability of CD8 T cells (58). In this setting, how cuproptosis or cuproptosis-inducing drugs affect the function of antitumor immune cells and the components of TME remains unclear. Further evidence from labs and beds is necessary to answer this question. There is hope that by inhibiting these immune-suppressive targets, HCC patients with high CRRS might enjoy an attenuated protumor impact of the TME components like CAFs. We also notice that CRRS showed a good predictability of response to sorafenib (**Figure 7B**). Studies found that sorafenib inhibits HCC by inducing ferroptosis (60, 61). Since cuproptosis is a programmed cell death distinct from ferroptosis (11) and HCC patients with a high CRRS showed resistance to cuproptosis, these patients might be suitable for treatment with sorafenib. Correspondingly, HCC patients with a low CRRS might represent a specific subgroup who are resistant to sorafenib. It is currently unclear whether cuproptosis affects HCC patients’ sensitivity to sorafenib or the other biological features of these patients, for example, lower mutant frequency of *TP53* (**Figure 5C**) (62, 63), contribute to such a resistance. Furthermore, the association between cuproptosis and the mitogen-activated protein kinase (MAPK) pathway or B-Raf proto-oncogene, serine/threonine kinase (BRAF) signaling should be investigated in the future, since these pathways play a crucial role in the acquired resistance to targeted therapy including sorafenib (64). This knowledge is of great importance for the future strategy of combined therapy, inducing cuproptosis or ferroptosis and targeting multiple pathways such as the rat sarcoma viral oncogene (RAS)/ Raf-1 proto-oncogene (RAF)/ mitogen-activated protein kinase kinase (MEK)/ extracellular signal-regulated protein kinase (ERK) pathway, in HCC and may lead to improved prognosis of these patients (64).

## Conclusions

**Figure 8:**
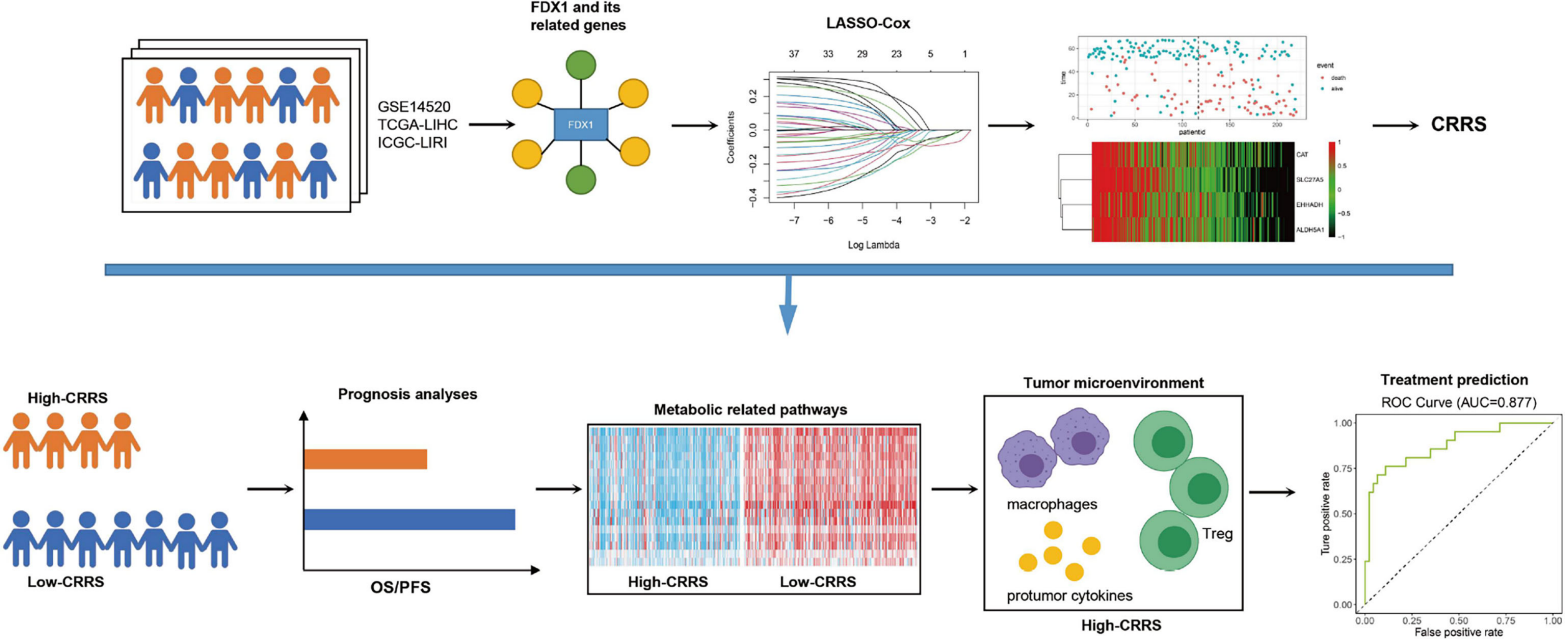
Summarization of this study. *FDX1* and its 36 related genes were identified in the HCC datasets. A novel quantified index, the CRRS, was constructed using the LASSO Cox regression model. HCC patients with high CRRS showed shorter survival time, lower enrichment in metabolic-related pathways, and high infiltration of protumor immune infiltrates. In addition, CRRS exhibited a good capability in predicting the responsiveness to sorafenib and the non-responsiveness to TACE.

In conclusion, the key regulator of cuproptosis, *FDX1*, was downregulated in HCC and its high expression was linked to poor prognosis of HCC patients. A novel CRRS, based on *FDX1* and its related genes, was constructed using the LASSO Cox regression model. HCC patients with a high CRRS showed shorter survival time, lower enrichment in metabolic-related pathways, and high infiltration of protumor immune cells. In addition, CRRS exhibited a good capability in predicting the responsiveness to sorafenib and the non-responsiveness to TACE (**Figure 8**).

## Data Availability Statement

The original contributions presented in the study are included in the article/**Supplementary Material**. Further inquiries can be directed to the corresponding author.

## Author Contributions

ZZ and XZ contributed equally to this work. The study was designed by ZS. Data analysis was carried out by ZS, ZZ, and XZ. Bioinformatics analysis was conducted by ZS and YW. XZ and YL provided useful advice to the analyses of the data. The manuscript was drafted by ZS and YW, and was revised by all authors before the final version was approved to be published.

## Funding

This work is supported by Natural Science Foundation of Hunan Province (Grant No.2021JJ31035).

## Conflict of Interest

The authors declare that the research was conducted in the absence of any commercial or financial relationships that could be construed as a potential conflict of interest.

## Publisher’s Note

All claims expressed in this article are solely those of the authors and do not necessarily represent those of their affiliated organizations, or those of the publisher, the editors and the reviewers. Any product that may be evaluated in this article, or claim that may be made by its manufacturer, is not guaranteed or endorsed by the publisher.
