# Supplementary material for: Cuproptosis-Related Risk Score Predicts Prognosis and Characterizes the Tumor Microenvironment in Hepatocellular Carcinoma
Source: Front Immunol. 2022 Jul 11;13:925618. doi: 10.3389/fimmu.2022.925618 (PMC9311491; doi:10.3389/fimmu.2022.925618)

Supplementary Figure legends:

Supplementary Figure 1: The expression of FDX1 in HCC. (A) The expression level of FDX1 across various types of normal tissues in the human protein atlas database. (B) The expression level of FDX1 across various types of tumors in the human protein atlas database. (C-D) The box and dot plot showing the expression of FDX1 between tumor and corresponding normal tissues in GSE76427 (C) and GSE25097 (D). (E) The optimal cutoff value of FDX1 in separating HCC patients with different prognosis in the TCGA-LIHC dataset. P values were shown as *p < 0.05, **p < 0.01, ***p < 0.001, and ****p < 0.0001.

Supplementary Figure 2: The construction of CRRS. (A-B) GO (A) and KEGG (B) analysis of FDX1 related genes. (C) FDX1 and its related genes were input into the LASSO Cox regression model, and the tuning parameter (λ) was calculated based on the partial likelihood deviance with 10-fold cross-validation. (D) The best cutoff value of CRRS in separating HCC patients with different prognosis in the GSE14520 dataset. (E) The boxplot showing the expression of cuproptosis-related genes in HCC patients from the high- and low-CRRS subgroups in the GSE14520 dataset. P values were shown as *p < 0.05, **p < 0.01, ***p < 0.001, and ****p < 0.0001.

Supplementary Figure 3: Features of CRRS-based classification. (A-B) GSEA of the high-CRRS group in the TCGA-LIHC cohort. (B) The box and dot plot showing TMB of HCC patients from the high- and low-CRRS subgroups. P values were shown as *p < 0.05, **p < 0.01, ***p < 0.001, and ****p < 0.0001.

Supplementary Figure 1


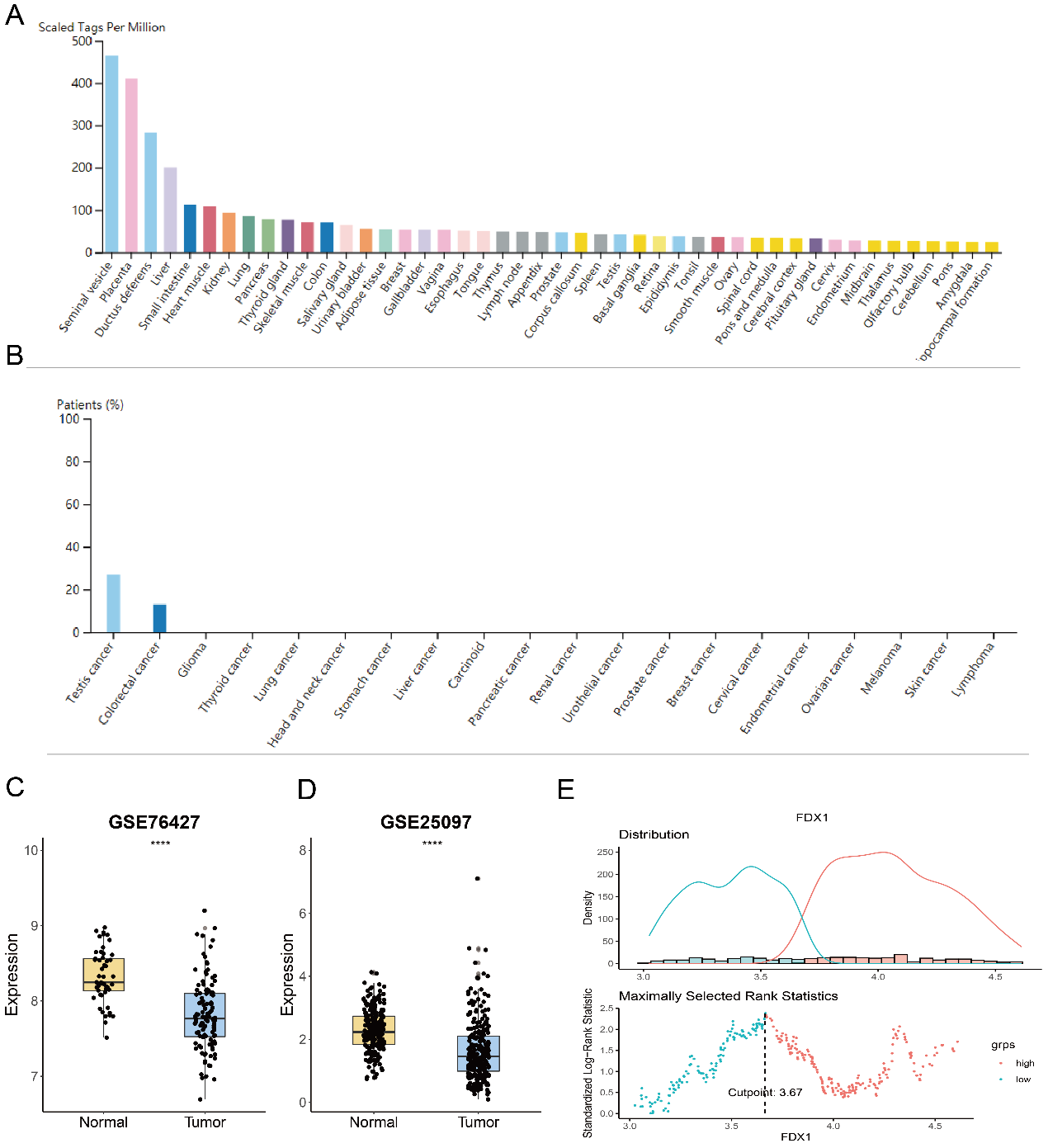


Supplementary Figure 2


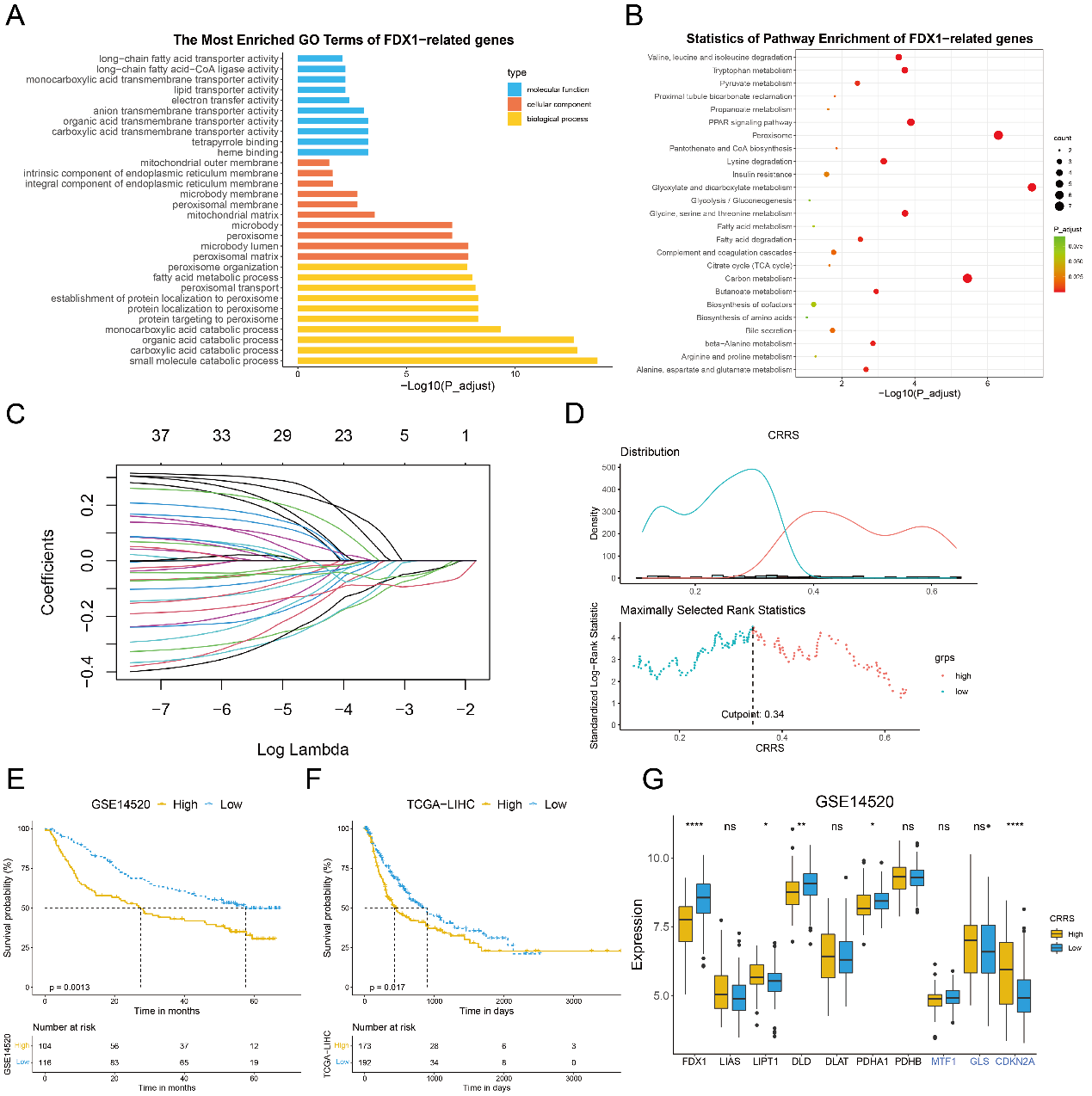


Supplementary Figure 3


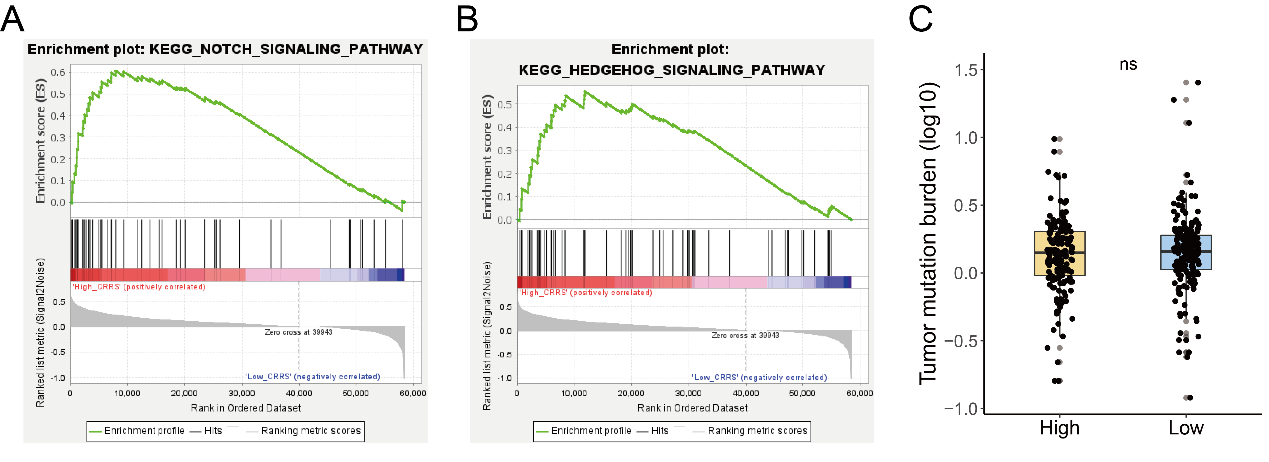

Supplement: Supplementary file 1 [file DataSheet_1.docx]
